# Cognitive Behavioural Therapy for Obsessive-Compulsive Disorder Related to the Fear of Internet Use: A Case Study

**DOI:** 10.7759/cureus.70584

**Published:** 2024-09-30

**Authors:** Ryotaro Ishikawa

**Affiliations:** 1 Clinical Psychology, Taisho University, Tokyo, JPN

**Keywords:** behavioural experiments, cognitive behavioural therapy, fear of internet use, obsessive-compulsive disorder, ocd with aggressive obsessions

## Abstract

Obsessive-compulsive disorder’s (OCD's) symptomatology appears to evolve with modern developments, with recent reports highlighting the influence of modern technologies (e.g., the Internet); for instance, some OCD cases are characterised by an excessive fear of Internet use. Meanwhile, cognitive behavioural therapy (CBT) is a form of psychotherapy that focuses on modifying dysfunctional thoughts, behaviours, and emotions, and has proven effective for various psychological issues, including OCD; however, research on OCD related to the fear of Internet use and its treatment remains limited. The present study presents a case of a patient who had been suffering from severe OCD for approximately one year, exhibiting a fear of using the Internet due to intrusive urges to send offensive messages (e.g., death threats) when near computers. This fear led to avoidance of Internet use and compulsive checking to ensure that Internet-connected devices were turned off. The treatment involved a combination of CBT and medication (fluvoxamine).

The case formulation focused on identifying thought-action-fusion-misinterpretations that exacerbate and maintain OCD (maladaptive appraisals about an increase in the probability of an unpleasant event by merely thinking about it); it also addressed cognitive and behavioural factors, including safety behaviours such as reassurance-seeking and thought suppression. To address and alter maladaptive interpretations and behaviours, CBT was implemented using various techniques, including behavioural experiments, which included the ‘on duty versus off duty’ tactic to relieve the patient from the responsibility of controlling aggressive thoughts, refraining from covering his computer or smartphone, intentionally looking at them frequently, and browsing various Internet forums and review sites. Through these experiments, the patient realised that his negative predictions were unfounded and that avoiding Internet use was unnecessary. The patient’s self-reported OCD symptoms, measured using the Japanese versions of the Yale-Brown Obsessive-Compulsive Scale and the Obsessive-Compulsive Inventory, decreased over the course of 10 sessions. This case study demonstrates that empirically supported CBT can effectively treat OCD related to aggressive obsessions and the fear of Internet use. Its limitations included that the patient was receiving both fluvoxamine and CBT (thus making it difficult to attribute the improvement solely to CBT) and that this study focused on a single patient setting (thereby making it difficult to generalize its findings).

## Introduction

Obsessive-compulsive disorder (OCD) has a lifetime prevalence of approximately 1-2% [1] and is categorised into four primary types: 1) contamination/cleaning, 2) doubt/checking, 3) symmetry/ordering, and 4) unacceptable thoughts [2,3]. The last category typically involves obsessions related to aggression or violence. Aggressive obsessions, sometimes referred to as harm OCD, are a common aspect of OCD, affecting approximately 58% of individuals with the disorder as one of their main symptoms [4]. These obsessions include distressing thoughts about violence and fears of doing something shocking or embarrassing, such as accidentally swearing, blurting out obscenities, or insulting someone. OCD compulsions can be either overt or covert [5]. Overt compulsions are observable, external behaviours (e.g., washing hands or checking locks), while covert compulsions are internal and less observable, such as attempting to counteract intrusive thoughts by thinking the opposite, a process known as mental ‘undoing’, and engaging in other thought-control behaviours such as thought suppression and distraction [5].

Individuals with OCD who experience unacceptable aggressive thoughts often engage in covert compulsions [6,7], such as mental rituals, and may also perform overt compulsions, such as reassurance-seeking [8,9]. In cognitive behavioural theory, these compulsive behaviours are referred to as safety behaviours [6,10,11]. Safety behaviours are strategies, either overt or covert, used in anxiety-provoking situations to reduce distress or prevent feared outcomes [12]. This term encompasses actions intended to protect against harm or responsibility for negative consequences, such as wearing good luck charms or gloves to avoid germ contamination. Although safety behaviours may provide short-term relief, they ultimately reinforce and sustain the intensity of obsessive thoughts and anxiety by creating the maladaptive belief that these behaviours are ‘necessary’ and that fear is permanently reduced through their use [6,12].

Considerations for treating OCD related to the fear of Internet use

The Internet is an integral part of modern life, with computers and digital devices being omnipresent. However, some individuals may develop Internet anxiety when using information technology (IT) applications [13]. A study reported a clinical case involving the fear of Internet use, where the patient experienced significant distress and anxiety about data safety and privacy while using the Internet and digital devices [14]. This anxiety led to avoidance of activities such as watching television, using email, withdrawing money through Internet banking, and relying on family members for help, resulting in significant dysfunction.

Another study described two cases of OCD characterised by an excessive fear of using smartphones and social media [15]. The first patient had obsessions related to losing control and accidentally posting a shameful or inappropriate reaction, such as violent or sexual content, on social networking sites (SNS). The second patient had obsessions regarding accidentally posting content that would be shameful or make her appear immoral. Another obsession involved the fear of inadvertently asking others to commit a crime. Both patients were treated with medication and cognitive behavioural therapy (CBT) [16]. In the previous case report, the authors noted that such cases are not uncommon and that the content of obsessions and compulsions often revolves around modern technologies [15]. Over time, the objects of fear for patients with OCD may evolve alongside technological advancements. A cross-sectional study also indicated that individuals with OCD are more affected by social media compared to those without OCD [17]. However, research specifically focused on OCD related to the fear of Internet use and its treatment is lacking. Furthermore, the previous case report did not provide detailed information on the CBT procedure, such as case formulation and behavioural experiments [15].

Cognitive behavioural therapy for OCD: efficacy and applicability

Behaviourally focused treatments, such as exposure and response prevention (ERP), have long been considered the gold standard for psychological treatment and have been tested in numerous randomised trials [18]. While ERP can be effective in alleviating a patient’s obsessions [19], early treatment trials that primarily utilised ERP strategies typically excluded participants with obsessions involving unwanted ego-dystonic intrusions of a sexual, aggressive, or blasphemous nature [20]. In cases where obsessions are the primary concern, treatment should focus on techniques specifically developed to address these types of obsessions [6,7].

With the development of cognitive theory in OCD [16,21], researchers and clinicians have discovered new cognitive-behavioural methods for treating OCD. Rachman refined the cognitive theory to specifically address unacceptable obsessions [6,22]. This treatment approach emphasises understanding the individual’s interpretations of their unwanted intrusive thoughts and helping them transform these interpretations into more adaptive and realistic alternatives. In addition, behavioural experiments are employed to test and modify maladaptive beliefs or behaviours, fostering the development of more adaptive alternatives through experiential learning [23]. The only randomised controlled trial specifically targeting participants with unacceptable obsessions tested Rachman’s model and found it to be highly effective (effect size d = 2.34), with results sustained over a one-year follow-up [7,24]. Although some studies have suggested that Rachman's model could be applicable in an Asian context [25], no studies have yet reported on the application of CBT based on Rachman’s model in Asian populations.

Components of CBT based on Rachman’s model

Rachman’s cognitive conceptualisation of primary obsessions posits that the experience of occasional intrusive and unwanted thoughts is a universal phenomenon [6]. The issue arises not from the presence of these intrusive thoughts but from the misinterpretation of their significance, which drives OCD. CBT, grounded in this theory, aims to reduce or eliminate a patient’s maladaptive appraisals of the personal significance of their obsessions. It also targets cognitive biases that lead to these maladaptive interpretations. Individuals with OCD may assign special importance to intrusive thoughts, experiencing what is known as thought-action-fusion (TAF) [22,26]. TAF is a psychological process where an individual believes that merely having a thought can influence one’s behaviour or the world around them. For example, someone with high TAF might believe that having a negative thought, whether intrusive or intentional, increases the likelihood of a negative event occurring. TAF has two dimensions: TAF-likelihood, which is the belief that merely thinking about an unpleasant event increases its probability (e.g., thinking about pushing an older adult onto a railway track is believed to actually increase the risk of doing so); and TAF-morality, which is the belief that having immoral thoughts is as unacceptable as actually committing immoral acts (e.g., thinking about pushing an older adult onto a railway track is considered almost as wicked as actually doing it) [6].

TAF plays an important role in the catastrophic interpretations of intrusive thoughts and is linked to OCD involving unacceptable obsessions or mental neutralisation [6,27]. Another key factor in dealing with intrusive thoughts is the effort to control them, such as through thought suppression. While thought suppression may provide temporary relief from distress, it can paradoxically increase the frequency of intrusive thoughts [11,27,28]. Thought suppression or control acts as a neutralising strategy to ameliorate the sense of responsibility for harm triggered by obsessional thought [29,30]. Beliefs about control over thoughts, specifically, the belief that preventing negative outcomes requires complete control over one’s thoughts, are central to OCD and underlie the misappraisal of thought recurrence or failed thought control [11,31]. In the CBT approach described by Rachman, therapists explain to their patients how certain cognitive biases (e.g., TAF and control over thoughts) contribute to an inflated sense of responsibility and promote attempts at thought suppression and neutralisation [6]. The therapists then work with the patient to develop an alternative, more accurate, and acceptable interpretation using cognitive-behavioural strategies such as behavioural experiments.

Our case report details the application of CBT techniques derived from Rachman’s theory in treating a patient with OCD in Japan who had aggressive obsessions and a fear of Internet use. Signed informed consent to publish the information was obtained from the client.

## Case presentation

Client characteristics and problems

Tom (pseudonym) is a 19-year-old college student who has never previously received psychiatric medication or psychotherapy. He first visited our private clinic due to a persistent avoidance of digital devices, such as smartphones, personal computers, and tablets. He was particularly fearful of using the Internet, as he experienced an overwhelming urge to send offensive messages or images when near or using these devices. His symptoms began at age 18 when he was browsing social media and Internet forums and came across a celebrity being bullied and insulted. He expressed his fear by saying, ‘If I were to post such offensive comments or something like death threats, I would be in serious trouble. I might even get sued, and the police might come to arrest me’. This fear deeply unsettled him.

As these thoughts became more frequent, Tom began to worry about actually writing death threats, slandering companies, and posting bullying messages online. He believed that ‘having the aggressive thought of posting things such as death threats increases the risk that I will actually do so’. Consequently, he became increasingly fearful, started avoiding the Internet, and began compulsively checking to ensure that his Internet-connected devices were turned off. His obsessive thoughts were so pervasive that he would hide his computer and smartphone by covering them with cloth or paper. When his family used smartphones near him, he would insist that they turn them off, forbidding their use around him, as he feared that seeing these devices might compel him to write something aggressive or offensive.

Tom’s symptoms worsened during the COVID-19 pandemic, particularly when remote classes became more frequent, and assignments had to be submitted online. When submitting an assignment, he worried that he might have written something offensive or inappropriate in the report. This led him to repeatedly check his work and hesitate to submit it. His obsessions extended beyond Internet use and into his daily life. For example, when he saw someone outside, he would experience obsessive thoughts and images, such as ‘I’m going to attack him using judo.’ Consequently, he began to avoid interpersonal relationships.

Assessment and treatment components

Tom was initially evaluated by a psychiatrist. Tom recognised his symptoms as indicative of a mental disorder and described them as ‘delusions’. He fully understood that his aggressive thoughts were inappropriate and immoral, and he was aware that his behaviours were irrational and his compulsive checking excessive. It was clearly determined that his intrusive thoughts were ego-dystonic, leading to the conclusion that these thoughts were not delusions. Based on the assessment results, the psychiatrist diagnosed Tom with OCD and prescribed fluvoxamine (100 mg). Even after a month of taking fluvoxamine, his symptoms were so severe that the psychiatrist recommended CBT. The author, a certified public psychologist with a PhD in Medicine, conducted the CBT sessions for Tom. The total treatment period was 12 weeks, with sessions held once every one to two weeks. Table 1 shows a summary of the CBT.

**Table 1:** Summary of the cognitive behavioural therapy

Session and focus	Content and session goals	Homework
Session 1: Psychoeducation and normalisation	To understand the cognitive theory of obsessive-compulsive disorder (OCD), including the following: Intrusive thoughts are not pathological. Suppression of intrusive thoughts is ineffective. Misinterpretation of intrusive thoughts exacerbates and maintains OCD.	Review the handout explaining the cognitive theory of OCD.
Sessions 2 and 3: Case formulation	To learn that safety behaviours, thought suppression, reassurance-seeking, and other neutralising behaviours may offer temporary relief but worsen obsessive thoughts over time.	Identify and record potential avoidance and safety behaviours in daily life. Review the case-formulation figure.
Session 4: Behavioural experiments addressing thought-action-fusion	To learn about thought-action-fusion and understand that thinking about offending others does not mean you will actually do it, through behavioural experiments.	Conduct behavioural experiments, such as imagining winning a lottery jackpot, breaking something, or practising Judo with others in daily life.
Session 5: Behavioural experiments of the ‘on duty vs. off duty’ tactic	To learn, through the behavioural experiments, that letting go of controlling aggressive thoughts does not lead to major trouble and that anxiety and urges related to aggressive obsessions disappear on their own without thought suppression.	Perform behavioural experiments using the ‘on duty vs. off duty’ tactic.
Sessions 6-9: Behavioural experiments using the Internet without safety behaviours	To test Tom’s negative prediction that using or seeing Internet devices will lead to posting offensive comments, and encourage a more adaptive interpretation, through behavioural experiments.	Conduct behavioural experiments by looking at smartphones or computers without covering them, and browsing various Internet forums and review sites.
Session 10: Psychoeducation on relapse prevention	To prevent recurrence by continuing behavioural experiments independently.	Continue to conduct behavioural experiments in daily life.

Prior to CBT, the severity of his symptoms was assessed using the Japanese version of the Yale-Brown Obsessive-Compulsive Scale (Y-BOCS) [32] and the Obsessive-Compulsive Inventory (OCI) [33]. His Y-BOCS scores were 18 for obsessions, 15 for compulsions, and 33 overall, indicating severe OCD. The OCI score of 58 suggested moderate OCD. Tom’s Global Assessment of Functioning (GAF) score was 50, reflecting significant symptoms or serious impairment in social, occupational, or academic functioning. His Clinical Global Impression-Improvement (CGI-I) score was 6, also indicating serious symptoms or impairment [34].

Session one: psychoeducation and normalisation

The therapist provided psychoeducation about OCD based on Rachman’s Cognitive Theory [6]. The session aimed to clarify that intrusive thoughts, which are commonly experienced by individuals with OCD, are not inherently pathological, and that having such thoughts is a normal part of the human experience. The therapist explained that attempts to suppress intrusive thoughts are generally ineffective and may, in fact, intensify their frequency and impact. Misinterpreting the meaning of such thoughts can further exacerbate and sustain OCD symptoms. The discussion also addressed how cognitive biases, such as TAF, and beliefs about controlling thoughts can worsen obsessive thoughts and lead to compulsive behaviours.

Sessions two and three: case formulation

In session two, Tom learned about the cognitive behavioural theory for OCD, including how avoidance and safety behaviours, such as compulsive checking, thought suppression, reassurance-seeking, and other neutralising behaviours, provide only temporary relief and can worsen obsessive thoughts over time [12]. For homework, Tom was asked to identify and list possible avoidance and safety behaviours in his daily life. Through this homework, Tom was able to identify his safety and avoidance behaviours (e.g., do not use Internet devices and cover them with a cloth to keep them out of sight).

**Figure 1:**
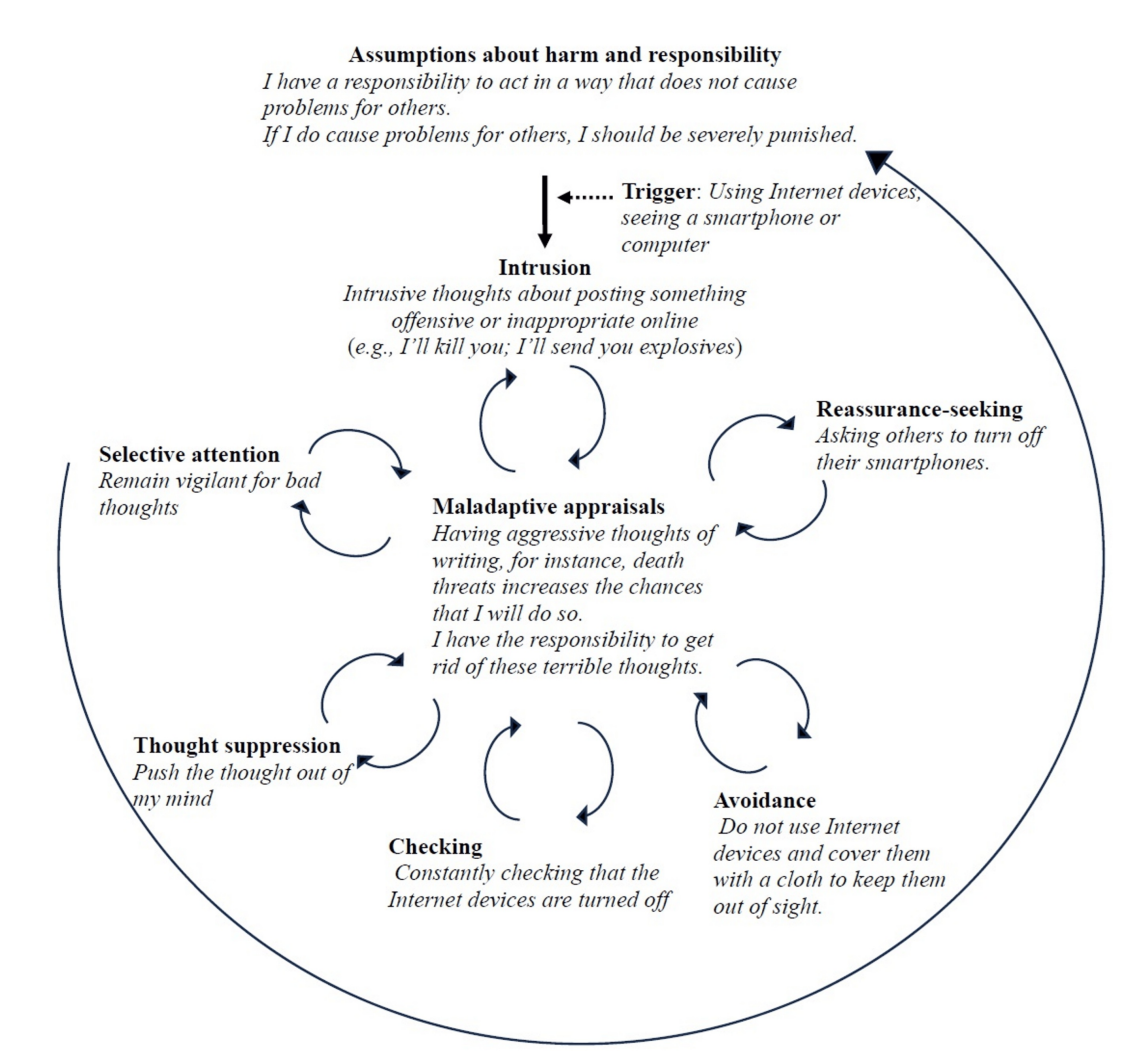
Case formulation Tom and the therapist created a vicious flower diagram to consolidate the various aspects of understanding obsessive-compulsive disorder (OCD). This figure illustrates the cognitive and behavioural factors that transform normal intrusive thoughts into OCD. By examining this diagram, Tom gained insight into the formation of the vicious cycle, which is crucial for understanding how OCD develops and persists. Image Credit: Author Ryotaro Ishikawa, based on the "vicious flower" model, introduced by Challacombe et al. [10] (p. 275)

In session three, the therapist provided psychoeducation to help Tom learn the basics of TAF. Subsequently, the therapist and Tom focused on identifying maladaptive interpretations based on TAF, selective attention, avoidance, thought suppression, and emotions that perpetuate the OCD cycle (Figure 1). A behavioural experiment, using the ‘white bear example’ [11], was conducted to demonstrate the paradoxical effects of thought suppression. Tom discovered that suppressing thoughts only increased the intensity, frequency, and impact. The case formulation highlighted that CBT could help modify his maladaptive responses, such as interpretation errors and safety behaviours, which were exacerbating his OCD.

Session four: behavioural experiments to address thought-action-fusion

To facilitate his engagement in the behavioural experiments to address TAF, the therapist first conducted an experiment involving the idea of causing positive events through mere thought [35]. Tom was asked to imagine winning a lottery jackpot and, in a slightly more negative scenario, to imagine causing the therapist’s computer to break. Following this, the therapist proposed a new experiment: ‘Let’s do a judo exercise, but only using our imaginations. We will simulate throwing me with judo techniques without physically touching each other. Let’s see what happens’. Tom agreed, and through the experiment, learned that merely thinking about offending others did not translate into actual behaviour, and he understood that thoughts were not as powerful as he had believed.

Session five: behavioural experiments with the ‘on duty versus off duty’ tactic

In this session, a behavioural experiment called the ‘on duty versus off duty tactic’ was conducted in the treatment room [6,36]. The purpose of this experiment was to compare periods of heightened responsibility and vigilance (‘on duty’) with periods of reduced responsibility and relaxation (‘off duty’). The goal was to help Tom learn to manage his sense of responsibility and safety behaviours.

The therapist instructed Tom to experience an ‘on-duty’ period for five minutes, during which he was required to be hyper-aware of all his thoughts and take full responsibility for controlling or suppressing any aggressive thoughts that might arise. Following this, Tom was guided through a five-minute ‘off-duty’ period’, where he was encouraged to relax, let go of the responsibility of controlling his thoughts, and engage in a restful activity, such as reading a magazine.

During the ‘on-duty’ period, Tom reported experiencing a high level of anxiety, worry, and distressing intrusive thoughts. However, during the ‘off-duty’ period, his anxiety and worry significantly decreased, and he realised that nothing problematic occurred even when he intentionally let go of control over his thoughts. This experiment helped Tom understand that taking ‘off-duty’ periods could effectively reduce the anxiety and distress associated with his obsessions.

As homework, the therapist and Tom planned a similar experiment. From 8:00 p.m. to 9:00 p.m., Tom would set aside ‘on-duty time’, during which he would engage in thought suppression and control of aggressive thoughts. The remainder of the evening would be treated as ‘off-duty time’, where he would avoid these controlling behaviours. Tom successfully completed this experiment at home, finding that even when he experienced aggressive obsessions during ‘off-duty’ time, he was able to resist excessive thought control, and no major issues arose. Through these exercises, Tom learned that releasing control over his aggressive thoughts allowed his anxiety and urges related to diminish naturally without the need for thought suppression.

Sessions six to nine: experiments using the Internet without safety behaviours

During Sessions six to nine, a series of behavioural experiments were conducted to challenge Tom’s negative prediction, ‘If I use or see Internet devices, I will post offensive comments’, and to help him develop a more adaptive interpretation. These experiments were first conducted in the counselling room with the therapist and then extended into Tom's daily life as homework.

The experiments involved various activities, such as not covering his computer or smartphone and attempting to look at them as frequently as possible, even when obsessive thoughts arose. Tom also browsed different Internet forums and review sites, intentionally avoiding any attempts to suppress or neutralise his obsessive thoughts. Additionally, he engaged in chatting with friends on SNS and played online games, interacting with opponents in real time. Through these experiments, Tom discovered that his negative predictions were unfounded. He realised that he no longer needed to avoid using Internet devices and felt confident that even if aggressive thoughts occurred while using these devices, they were merely thoughts and did not influence his actions.

Session 10: the final session

By the time of the final session, Tom had resumed attending university and was able to use the Internet to complete assignments. In this session, the therapist provided psychoeducation on relapse prevention, reviewed the case formulation, and revisited the series of behavioural experiments. The therapist emphasised the importance of Tom remaining mindful of his OCD symptoms and continuing to conduct behavioural experiments to prevent a recurrence of his symptoms.

Follow-up

Follow-up sessions lasting 20 minutes were held every one to two months after the completion of CBT treatment. During these sessions, it was confirmed that Tom’s OCD symptoms did not recur. Fluvoxamine, which had been administrated for approximately five months, was discontinued after the first follow-up session, as the psychiatrist determined that his symptoms had remained stable. Tom expanded his range of activities, found his social life at the university more enjoyable, and even attended a pop concert. Moreover, he was able to work part-time without experiencing significant anxiety.

Evaluation of treatment outcomes

**Figure 2:**
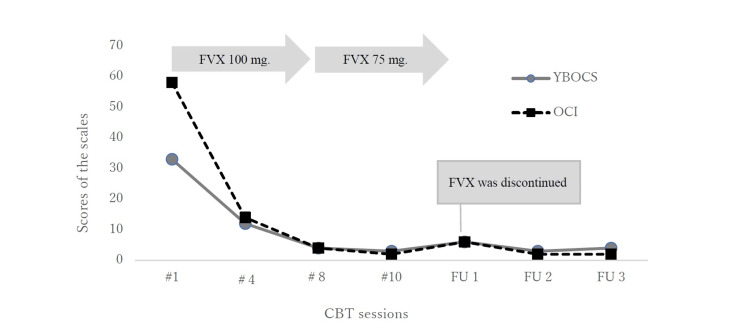
Total scores for the Yale-Brown Obsessive Compulsive Scale (Y-BOCS) and Obsessive-Compulsive Inventory (OCI) during the 10 treatment sessions and at follow-up Y-BOCS: Self-report version of the Yale-Brown Obsessive-Compulsive Scale (Japanese version); OCI: Obsessive-Compulsive Inventory (Japanese version); CBT: Cognitive Behavioural Therapy; FU: Follow-up; FVX: Fluvoxamine The CBT treatment spanned 12 weeks held every one to two weeks. Follow-up sessions, lasting 20 minutes each, were conducted approximately every one to two months after the CBT treatment. Fluvoxamine was prescribed starting one month before CBT and continued for five months until the first follow-up. The Y-BOCS Japanese version rates the severity of OCD on a scale of 40 points: less than 8 indicates no OCD, 8-15 denotes mild, 16-23 signifies moderate, 24-31 represents severe, and 32-40 indicates very severe OCD. An OCI total score of 42 or higher suggests the presence of OCD.

In the course of the CBT, Tom developed a new adaptive interpretation: ‘Having aggressive and intrusive thoughts is a normal phenomenon, holds no inherent meaning, and will disappear on its own without the need for control’. Self-reported OCD symptoms were measured using the Y-BOCS and OCI. Over the 10 sessions, Tom’s total Y-BOCS score decreased from 33 to 3, and his total OCI score dropped from 58 to 2. His GAF score increased from 50 to 90, reflecting minimal or no symptoms, and the CGI severity score decreased from 6 to 1 (Figure 2).

The CBT treatment took place during Tom’s university spring break, and by session nine, when classes resumed, he was able to attend without any issues and use the Internet for assignments. Tom also spent time with friends, chatted on social media, and played online games. He expressed confidence in his ability to manage potential OCD symptoms, even if aggressive intrusive thoughts occurred. As a third-party evaluation, Tom's mother reported during the follow-up interview that his symptoms remained stable at home and that his reassurance-seeking behaviours, such as asking others to turn off their smartphones, had completely ceased. The psychiatrist also noted that the medication was discontinued due to the stability of Tom’s symptoms.

## Discussion

This study is groundbreaking as the first, to the best of our knowledge, to apply CBT based on Rachman’s model to a patient within an Asian context. As research continues to demonstrate the effectiveness of CBT, this approach could offer a valuable treatment option for Asian patients experiencing obsessions, as well as for their clinicians. In this study, the scores of the Y-BOCS and OCI decreased considerably over 10 sessions of CBT. These results show that CBT treatment using Rachman’s model was effective for OCD regarding the fear of internet use. CBT could lead to the development of self-mastery, insights, and life skills that medication alone cannot provide. For instance, through CBT, Tom could learn that aggressive intrusive thoughts are not pathological and relinquish control over them, allowing his anxiety and urges related to compulsive checking to diminish naturally without the need for thought suppression.

Learning these adaptive beliefs and behaviours likely contributed to the significant improvement in his Y-BOCS and OCI scores. A key advantage of CBT is that it empowers patients to learn coping strategies for managing OCD independently. Therefore, individuals who successfully undergo CBT are better equipped to handle intrusive thoughts or OCD-like experiences even after remission, reducing the risk of relapse. Indeed, some clinical studies indicated that CBT was effective in preventing relapse for OCD [37,38]. Another factor that may have contributed to Tom’s improvement was the support of his mother. She attended several of his CBT sessions and became familiar with the behavioural experiment procedures. In his daily life, when Tom engaged in reassurance-seeking behaviours, such as asking her to turn off her smartphone, his mother suggested the ‘off-duty’ tactic. Tom reported that her support was effective in helping him discontinue these behaviours. In addition, the treatment coincided with a long holiday from university, providing Tom with a calm environment where he could focus on his treatment.

This case study is also the first, to the best of our knowledge, to detail the specific procedures of CBT for OCD related to the fear of Internet use. The inability to use the Internet as a result of OCD can greatly impede a person’s social functioning and quality of life, as it did with Tom, who was unable to attend university. One study noted that such cases are not uncommon [15], yet research on this type of OCD is limited. This may be because patients with these concerns often worry about data privacy, making them hesitant to share their information publicly. Unlike typical patients with OCD, Tom had no prior knowledge of OCD or its treatments, largely because his symptoms were not typical, and his fear of using the Internet left him isolated and unable to research his condition. Fortunately, Tom's mother researched CBT on his behalf and scheduled an appointment for him. However, other patients with similar fears may remain isolated and unable to access treatment due to their Internet-related anxieties. For instance, in many clinical settings, appointments are made online, which could pose a barrier to these individuals. Clinicians and researchers need to be mindful of the challenges faced by people with mental disorders who are apprehensive about using the Internet.

Additionally, the author recommends incorporating Rachman's psychoeducation to normalise obsessions and highlight the paradoxical effects of thought suppression as adjuncts to ERP-centred treatments. While incidental or occasional psychoeducation on normalising aggressive thoughts and the paradoxical effects of thought suppression may have been included in ERP treatments, systematically implementing this aspect of Rachman's CBT protocol could enhance the treatment of obsessions. This approach is particularly beneficial for therapists who are new to CBT or work in cultural contexts where CBT is less established. In addition, to the best of our knowledge, no randomised controlled trials have been conducted in Asia to evaluate the effectiveness of treatments for obsessions. Future studies could examine the efficacy of Rachman’s CBT for treating obsessions in Asian patients through randomised controlled trials.

Limitations

Although the present study reveals important findings, it has several limitations. First, Tom was receiving both fluvoxamine and CBT, so it is unclear whether the improvement in his symptoms can be attributed solely to CBT. Further empirical studies are needed to clarify the extent to which CBT alone is effective in treating OCD related to the fear of the Internet. However, a previous clinical trial over 12 weeks using a Japanese sample of individuals with OCD showed that while fluvoxamine effectively reduced Y-BOCS scores, behavioural therapy was more effective [39]. Considering such a result, it is possible that the effect of CBT may be a major factor in the improvement in Tom's symptoms over the course of approximately 12 weeks in our case report. In addition, recent systematic reviews and meta-analyses suggest that for severe OCD, a combination of medication and psychological treatment, such as CBT, is recommended [38,40]. Given this research background, introducing CBT alongside medication is considered appropriate.

Second, this case report focuses on a single patient setting, making it difficult to generalize the findings. Consequently, in this case report, it is impossible to completely rule out the possibility that Tom's improvement was a placebo effect. Future studies with larger sample sizes would be beneficial in building on the knowledge gained from this case study. In addition, due to Tom's request, primarily because of the cost, follow-up CBT sessions were only short-term. Therefore, research is needed to verify the long-term effectiveness of CBT in diverse experimental settings.

Finally, given the lack of research on the prevalence and treatment challenges of OCD associated with Internet-related fears, future directions should include quantitative psychopathological studies. Adding specific items about obsessions and compulsions related to the fear of Internet technology to the Y-BOCS, or developing a new self-report scale to assess these symptoms, would be valuable. As suggested by one study [15], such a scale should include items on obsessions (e.g., fear of posting something offensive or embarrassing, fear of breaches of privacy, or fear of cyberattacks), compulsions (e.g., checking posts, ensuring devices are turned off, or seeking reassurance), and avoidance behaviours (e.g., avoiding Internet use). These self-report scales would contribute to the literature on OCD with Internet-related fears, raise awareness among clinicians, and assist in planning suitable assessments and treatment targets. In addition, young people may be more susceptible to developing OCD related to the fear of using the Internet. Both our case report and a previous similar case report involved young patients [15], which may be due to their frequent use of SNS and Internet forums. This increased usage makes them more aware of the various issues and risks associated with these platforms. Furthermore, young individuals who are particularly sensitive to anxiety may be even more wary of these risks.

## Conclusions

Although research on OCD related to the fear of Internet use and its treatment is limited, this study is the first, to the best of our knowledge, to detail the specific CBT procedure, such as behavioural experiments, for this type of OCD. In today’s world, where patients have unlimited access to smartphones and social media, which can potentially trigger obsessions and compulsions throughout the day, it is important for clinicians and researchers to be aware of and address the concerns of patients who fear using the Internet. As a limitation, the patient was receiving both fluvoxamine and CBT, making it difficult to attribute the improvement solely to CBT. In addition, this case report focuses on a single patient setting, making it difficult to generalize the findings. Further studies with larger sample sizes are needed to clarify the effectiveness of CBT alone in treating this type of OCD.
